# Persistent Hyperplastic Primary Vitreous With Complete Retinal Detachment in an Infant: Imaging Characteristics and Clinical Management

**DOI:** 10.7759/cureus.96420

**Published:** 2025-11-09

**Authors:** Shrinivas Radder, Nivedita Radder

**Affiliations:** 1 Diagnostic Radiology and Pediatric Radiology, University of Arkansas for Medical Sciences, Arkansas Children's Hospital, Little Rock, USA; 2 Diagnostic Radiology, University of Arkansas for Medical Sciences, Little Rock, USA

**Keywords:** leukocoria, magnetic resonance imaging, microphthalmos, pediatric ophthalmology, persistent fetal vasculature, persistent hyperplastic primary vitreous, retinal detachment

## Abstract

Persistent hyperplastic primary vitreous (PHPV), also known as persistent fetal vasculature, is a rare congenital ocular developmental anomaly that can lead to significant visual impairment if not promptly recognized and managed. We present a case of a one-month-old male infant who presented with decreased opening of the left eye and leukocoria. Clinical examination revealed a white pupillary reflex with absent visual fixation. Ophthalmoscopic evaluation demonstrated a grey retrolental mass with prominent vascular loops. B-scan ultrasonography identified a characteristic retrolental mass connected to the optic nerve head by a thin stalk. Magnetic resonance imaging confirmed the diagnosis, revealing microphthalmos with a V-shaped retrolental fibrovascular mass extending to the optic nerve head, accompanied by complete retinal detachment and vitreous hemorrhage. The absence of calcification helped differentiate this condition from retinoblastoma. Due to the severity of structural abnormalities and poor visual prognosis, enucleation with serial conformer placement was performed. This case highlights the importance of multimodal imaging in diagnosing PHPV and emphasizes the role of MRI in surgical planning and excluding associated intracranial anomalies.

## Introduction

The discovery of a white pupillary reflex in an infant invariably triggers concern among parents and clinicians alike. While retinoblastoma remains the most feared cause of leukocoria, persistent hyperplastic primary vitreous (PHPV) represents the second most common etiology, accounting for approximately 5% of childhood blindness in developed nations [1]. This congenital ocular anomaly results from the failure of the embryonic hyaloid vascular system to regress appropriately during fetal development, leading to a spectrum of intraocular abnormalities that can severely compromise visual function [2].

Our understanding of PHPV has evolved considerably since its initial description. The condition typically presents unilaterally in 90% of cases and demonstrates variable clinical severity ranging from isolated posterior lens opacities to severe microphthalmos with complete retinal detachment [3]. Early recognition and accurate diagnosis are crucial, as the visual prognosis and management strategies differ significantly from those of other causes of leukocoria, particularly retinoblastoma.

We present a case of severe unilateral PHPV in a one-month-old infant, emphasizing the complementary roles of ultrasonography and magnetic resonance imaging in establishing the diagnosis and guiding clinical management.

## Case presentation

A previously healthy one-month-old male infant was brought to our pediatric ophthalmology clinic by his concerned parents, who noticed that their son's left eye appeared smaller and "did not open properly" since birth. The pregnancy had been uncomplicated, with the infant delivered at full term via spontaneous vaginal delivery. There was no history of prematurity, oxygen therapy, or maternal infections during pregnancy. Family history was unremarkable for ocular disorders or malignancies.

On examination, the infant appeared well-nourished and developmentally appropriate for age. The right eye demonstrated a normal red reflex with appropriate visual tracking. In contrast, the left eye exhibited microphthalmos with a striking white pupillary reflex (leukocoria). The infant showed no visual fixation or following response in the affected eye. Intraocular pressure measurement was technically challenging, but appeared elevated in the left eye.

Slit-lamp biomicroscopy of the left eye revealed a shallow anterior chamber with elongated ciliary processes. The lens appeared dysplastic with posterior displacement. Most remarkably, pale retinal tissue was visible directly behind the lens, suggesting severe anterior displacement of the retina. Dilated fundoscopy, though limited by the media opacity, revealed a gray-white retrolental mass with prominent, tortuous vascular loops visible in the temporal and superior nasal quadrants.

Given these concerning findings, immediate imaging was pursued. B-scan ultrasonography demonstrated a characteristic funnel-shaped retrolental echogenic mass extending from the posterior lens surface to the optic nerve head, connected by a thin echogenic stalk representing the persistent hyaloid artery. The globe measured 16 mm in axial length, confirming the diagnosis of microphthalmos.

**Figure 1:**
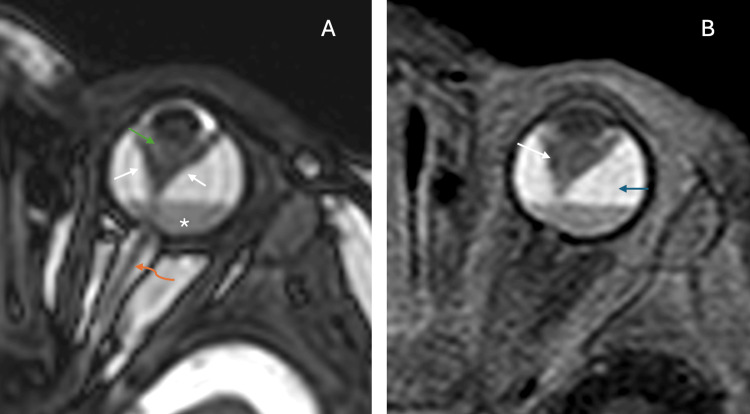
Axial magnetic resonance imaging of the orbits in a one-month-old infant with persistent hyperplastic primary vitreous (PHPV). (A) Axial heavily T2-weighted image (TR/TE 2500/120 ms, 3-mm slice thickness) demonstrates marked left microphthalmos with an abnormal funnel-shaped hypointense structure within the vitreous cavity. The detached retina forms a characteristic V-shaped configuration (white arrowheads) with its apex converging posteriorly at the optic disc. An ill-defined hypointense structure (green arrow) extends from the posterior lens surface to the optic nerve head, representing the persistent hyaloid artery within Cloquet's canal. Heterogeneous signal intensity within the vitreous cavity suggests hemorrhage with fluid-fluid levels (indicated by an asterisk). The lens appears dysplastic and has irregularity along the posterior margin. The left optic nerve is hypoplastic (curved orange arrow). (B) Axial post-contrast T1-weighted fat-saturated image (TR/TE 516/12 ms, 3-mm slice thickness) reveals enhancement of the retrolental fibrovascular tissue (white arrow) and the persistent hyaloid stalk. The subretinal space demonstrates intrinsic T1 hyperintensity (blue arrow) consistent with proteinaceous fluid or hemorrhage, without significant enhancement. No considerable enhancement noted elsewhere.

Magnetic resonance imaging was subsequently performed to better characterize the intraocular pathology and exclude the possibility of retinoblastoma. On a heavily T2-weighted sequence (Figure 1A), the left globe demonstrated severe microphthalmos with an axial length of 16 mm compared to 21 mm on the right. A characteristic funnel-shaped hypointense structure was identified within the vitreous cavity, formed by the detached and folded retinal leaves, creating a distinctive V-shaped configuration with the apex pointing posteriorly toward the optic disc. A thin, linear hypointense structure extended through the center of this funnel from the posterior lens capsule to the optic nerve head, representing the persistent hyaloid artery traversing through Cloquet's canal. The vitreous cavity showed heterogeneous T2 hypointense areas and fluid-fluid levels, consistent with hemorrhage of varying ages. The lens appeared dysplastic with abnormal morphology. The left optic nerve seemed to be hypoplastic. Post-contrast T1-weighted fat-saturated image (Figure 1B) demonstrated enhancement of the retrolental fibrovascular mass and the persistent hyaloid stalk, confirming their vascular nature. The subretinal space showed intrinsic T1 hyperintensity without significant enhancement, suggesting proteinaceous fluid or subretinal hemorrhage rather than active vascular proliferation. Crucially, no abnormal enhancement suggestive of retinoblastoma was identified, and no calcifications were present on any sequences. The right orbit and globe appeared entirely normal, as did the brain parenchyma (not included in the image).

Based on the clinical and imaging findings, a diagnosis of severe posterior PHPV with complete retinal detachment was established. After extensive discussion with the parents regarding the poor visual prognosis and risk of complications, including glaucoma and phthisis bulbi, the decision was made to proceed with enucleation. The infant underwent uncomplicated enucleation of the left globe with placement of an orbital implant. Serial conformers were subsequently fitted to promote normal orbital growth. Histopathological examination confirmed the diagnosis, demonstrating persistent primitive vitreous tissue with hyaloid vasculature and total retinal detachment.

## Discussion

This case exemplifies the severe end of the PHPV spectrum, where early and extensive developmental disruption results in profound structural abnormalities incompatible with visual function. The pathophysiology of PHPV centers on the failure of the primary vitreous and its associated hyaloid vasculature to undergo programmed regression during the third trimester of fetal development [4]. This persistence of embryonic tissue can manifest in various forms, traditionally classified as anterior, posterior, or combined, with our case representing severe posterior involvement.

The imaging findings in our patient were pathognomonic for PHPV. The V- or funnel-shaped configuration seen on MRI, formed by the detached and anteriorly displaced retina, represents a classic finding in posterior PHPV [5]. The persistent hyaloid artery, visualized as a linear structure extending from the optic disc to the posterior lens, serves as a virtually diagnostic feature when present. The associated microphthalmos, observed in approximately 80% of PHPV cases, further supported the diagnosis [6].

The presence of vitreous hemorrhage with fluid-fluid levels, as demonstrated in our case, is a well-recognized complication of PHPV resulting from the fragility of the persistent fetal vasculature. The intrinsic T1 hyperintensity in the subretinal space without enhancement suggests chronic proteinaceous or hemorrhagic fluid accumulation rather than active neovascularization, an important distinction when considering differential diagnoses.

The differential diagnosis of leukocoria in infancy encompasses several sight-threatening and life-threatening conditions. Retinoblastoma remains the primary concern, accounting for the most common intraocular malignancy in children. The absence of calcification in our case, while not absolutely excluding retinoblastoma, made this diagnosis highly unlikely, as over 90% of retinoblastomas demonstrate calcification on imaging [7]. Additionally, retinoblastomas typically show marked enhancement with a solid appearance, contrasting with the linear enhancing structures seen in PHPV. Coats disease, another crucial differential consideration, presents typically in older children and rarely causes microphthalmos. The presence of a central vitreous stalk effectively excluded this diagnosis. Retinopathy of prematurity was ruled out by the absence of prematurity and the unilateral presentation.

The management of PHPV depends critically on the severity of involvement and the potential for visual rehabilitation. In cases with mild anterior involvement and clear visual axis, early cataract extraction with aggressive amblyopia therapy may preserve functional vision [8]. However, in severe cases like ours, with complete retinal detachment, microphthalmos, and absent visual potential, enucleation becomes necessary to prevent complications and address cosmetic concerns [9]. The placement of an orbital implant and serial conformers is essential to promote normal orbital growth and facial symmetry.

Recent molecular studies have begun to elucidate the genetic basis of PHPV, with mutations in genes regulating vascular development, including the Norrie disease gene (NDP) and FZD4, identified in some cases [10]. While most cases occur sporadically, these discoveries raise the possibility of genetic counseling for affected families and may ultimately lead to novel therapeutic approaches.

The characteristic appearance of PHPV has been well-documented in multiple imaging studies. Mafee et al. initially described the funnel-shaped mass occupying the retrolental space and Cloquet canal on CT imaging [11]. This finding was further validated by subsequent MRI studies, which demonstrated superior soft tissue contrast [12]. Kadom and Sze emphasized the role of MRI in differentiating PHPV from other causes of leukocoria, particularly its ability to demonstrate the absence of calcification that distinguishes it from retinoblastoma [13]. Additionally, the use of high-resolution 3D MRI sequences, such as constructive interference in steady state (CISS), has enhanced visualization of delicate intraocular structures, allowing for better delineation of the persistent hyaloid vessel and associated abnormalities [14].

Kaufman et al. noted that hemorrhage within the vitreous cavity occurs in up to 30% of PHPV cases and may contribute to poor visual outcomes [15]. Smirniotopoulos et al. provided a comprehensive review of the differential diagnosis of leukocoria, emphasizing that the presence of microphthalmos strongly favors PHPV over retinoblastoma, which typically presents with a normal or enlarged globe [16]. Apushkin et al. highlighted that Coats disease characteristically shows subretinal exudates with high lipid content that appear hyperintense on both T1 and T2-weighted images without enhancement, distinguishing it from the enhancing fibrovascular tissue seen in PHPV [17].

The association of PHPV with cerebral abnormalities, as seen in our case, has been increasingly recognized in the literature. Walsh et al. reported that up to 15% of patients with PHPV have associated systemic or neurological abnormalities, including microcephaly, developmental delay, and seizure disorders [18]. The cerebellar hypoplasia and gyration anomaly observed in our patient are concerning for a syndromic association, possibly Walker-Warburg syndrome or other congenital muscular dystrophy-related conditions [19].

Anteby et al. reported visual acuity of 20/40 or better in 25% of eyes with anterior PHPV treated with early surgical intervention [20]. Sanjari et al. demonstrated improved cosmetic outcomes with early orbital rehabilitation following enucleation [21]. Prasov et al. identified mutations in ATOH7, a gene crucial for retinal ganglion cell development, in familial cases of PHPV [22]. Pendergast et al. reported that approximately 10% of PHPV cases have an identifiable genetic cause, suggesting the importance of genetic counseling for affected families [23]. Long-term follow-up studies have provided valuable insights into the natural history of PHPV. Dass and Trese reported that eyes with PHPV that were not enucleated had a 40% risk of developing glaucoma and a 20% risk of developing phthisis bulbi within 10 years [24].

## Conclusions

Persistent hyperplastic primary vitreous remains an important cause of infant leukocoria that requires prompt recognition and appropriate management. This case highlights the importance of multimodal imaging, particularly the complementary roles of ultrasonography and MRI, in establishing a diagnosis and differentiating PHPV from retinoblastoma. The characteristic imaging findings, including the V-shaped retinal detachment, persistent hyaloid vessel, and associated hemorrhage, when combined with the absence of calcification, allow confident diagnosis. While the visual prognosis in severe cases remains poor, early diagnosis allows for timely intervention to prevent complications and optimize cosmetic outcomes. As our understanding of the molecular mechanisms underlying PHPV continues to evolve, future therapeutic strategies may hold promise for preserving vision in affected infants.
